# Sildenafil Transiently Delays Early Alveolar Healing of Tooth Extraction Sockets

**Published:** 2017-05-03

**Authors:** Elysse Orchard, Wanda Green, Renjith Parameswaran Nair, Fleurette Abreo, Gulshan Sunavala-Dossabhoy

**Affiliations:** 1 Department of Animal Resources, Louisiana State University Health Sciences Center, 1501 Kings Highway, Shreveport, Louisiana 71130, USA; 2 Department of Cellular Biology and Anatomy, Louisiana State University Health Sciences Center, 1501 Kings Highway, Shreveport, Louisiana 71130, USA; 3 Department of Biochemistry and Molecular Biology, Louisiana State University Health Sciences Center, 1501 Kings Highway, Shreveport, Louisiana 71130, USA; 4 Department of Pathology, Louisiana State University Health Sciences Center, 1501 Kings Highway, Shreveport, Louisiana 71130, USA

**Keywords:** PDE5, cGMP, Bone regeneration

## Abstract

Bone is a unique tissue that has the ability to repair itself and return to full function. Bone regeneration is a well synchronized biological process that recapitulates embryonic bone development. The establishment of a functional vascular supply has been shown to be essential for proper ossification of newly deposited bone, and impaired angiogenesis as in advanced age, diabetes, and anti-cancer treatments affect bone repair. Endothelial Guanosine, 3′, 5′-Cyclic Monophophate(cGMP) is known to support angiogenesis, and sildenafil, a Phosphodiesterase 5 (PDE5) antagonist, prevents cGMP hydrolysis and thereby, promotes the formation of new blood vessels. Since the development of functional vascular networks is critical to bone repair, we investigated the effects of sildenafil on early alveolar bone regeneration following exodontia. Our results demonstrate that per-oral administration of sildenafil (10 mg/kg/day) in rats delays the dissolution and replacement of the sanguine clot with granulation tissue. As a result, the number of replicating cells, a hallmark of regenerating tissue, observed on day 4 was remarkably lower in sildenafil-treated animals than their control counterparts (mean±SD; control: 47.35±9.21; sildenafil: 11.47±5.14). Similarly, cells expressing transcription factor Cbfa-1/Runx2 and osteopontin, markers of differentiating osteoblasts, were fewer in treated animals (mean±SD; control: 83.18 ± 4.60; sildenafil: 13.77 ± 4.63). Treatment with hydrolysis-resistant cyclic GMP (cGMP) showed findings similar to sildenafil-treated animals suggesting a negative impact of cGMP on early inflammatory phase of bone healing. However, histological differences were not significant between the 2 groups on day 8. Based on these findings, we conclude that sildenafil temporarily retards early events in alveolar bone healing.

## Introduction

Bone regeneration is a biological process that involves a cascade of well-synchronized responses essentially comprised of three overlapping stages namely, inflammatory, reparative, and remodeling. Activation of endothelial cells and the sprouting of new capillaries from pre-existing vasculature is a process referred to as angiogenesis. Angiogenesis is critical for bone healing [1,2], and angiogenesis inhibitors have been shown to block outgrowth of new blood vessels and delay calcification of newly deposited bone at fracture sites [3–5]. Embryonic development of facial bones and flat bones of the skull occurs by intramembranous ossification, and bone regeneration within the alveolus after tooth extraction recapitulates embryonic development. The fibrin clot formed soon after extraction undergoes organization and is replacement with a stem cell-rich connective tissue. In response to inflammatory signals, stem cells undergo differentiation to cells of angiogenic and osteogenic lineages, and cellular events ultimately culminate in bone deposition and maturation. The formation of new blood vessels is intimately connected to bone repair, and impaired angiogenesis as in old age or diabetes, or radiotherapy delay healing. The role of endothelial cyclic guanosine monophosphate (cGMP) in modulating vascular tone and permeability during angiogenesis has been demonstrated before [6,7]. Phosphodiesterase 5 (PDE5) is a potent enzyme that regulates intracellular cGMP levels by promoting the conversion of cGMP to GMP. PDE5 antagonists such as sildenafil that suppress the hydrolysis of cGMP increase intracellular cGMP pool. Sildenafil treatment has been shown to increase angiogenesis and improve soft tissue survival after embolic stroke, ischemic limb pathology, and random-pattern flap grafts in animals [8–11]. Moreover, nitric oxide (NO) is known to interact with soluble guanine cyclase to stimulate the production of cGMP, and NO was also found to improve tissue recovery after ischemic pathology [12,13]. NO is a well-documented potent stimulator of cell proliferation and angiogenesis during wound healing[14–16], and wound repair is impaired and bone formation reduced in endothelial nitric oxide synthase (eNOS)-deficient animals and in animals administered nitric oxide synthase (NOS) inhibitors [17,18]. Osteoblasts of eNOS knockout animals demonstrated a delay in bone maturation, and exogenous supplementation of NO upregulates genes involved in osteoblast differentiation and matrix mineralization [15]. Based on the involvement of NO/cGMP pathway in bone regeneration, the aim of the study was to investigate the effects of PDE5 inhibition on alveolar bone healing after tooth extraction.

## Materials and Methods

### Reagents

Sildenafil was a kind gift from Pfizer (New York, NY), whereas 8-bromo-cGMP was purchased from Calbiochem. Primary antibodies, anti-Ki67, anti-von Willebrand factor, anti-Osteopontin and anti-Runx2 were purchased from Abcam. The HRP-conjugated secondary antibodies and HRP-detection kit were obtained from Biogenex.

### Endothelial tube formation

Human umbilical vein endothelial cells, HUVEC, were grown in Dulbecco’s Modified Eagles Medium (DMEM) that was supplemented with 5% FBS, antibiotic-antimycotic (Invitrogen) and minimal essential amino acids (Invitrogen). Capillary tube formation was conducted in 6 well culture plates coated with low growth factor matrigel (BD Biosciences). HUVEC cells were seeded at 1×10^4^ cells/cm^2^ in DMEM with 5% FBS with, or without, sildenafil (100 nM) and cells were imaged at various times. Capillary tube formation was quantified in 3 random areas of the well and the length of cords between cells was measured using ImageJ Angiogenesis Analyzer.

### Animals and experimental procedure

Forty outbred male Wistar rats (Harlan/Envigo) weighing approximately 250 gm were housed under 12 h dark and light cycle with free access to food and water. Experiments were performed under the aegis of the LSUHSC Animal Care and Use Committee, and they conformed to the guidelines for the care and use of laboratory animals. Animals were anesthetized with intramuscular administration of ketamine chloride (42mg/kg BW), xylazine (8mg/kg BW), and acepromazine (1.4 mg/kg BW), and the left mandibular first molars were luxated. Bleeding was stanched by pressure at the extraction site. Animals were kept warm during recovery and were housed based on treatment. Sildenafil was dissolved in sterile water, and 200 μl of sildenafil (10 mg/kg/day; divided into 2 doses administered 10 h apart) or water was administered by gavage each day starting the day of tooth extraction. Due to the 8-fold increase in plasma clearance rate and a short half-life of sildenafil in rodents (>1 hour in mice versus 4 hours in humans; [19,20]), the dosage of sildenafil was adjusted to achieve therapeutic dosing comparable to human. Animals were euthanized under anesthesia by carbon dioxide inhalation on day 2, 4, or 8, and the left segment of the mandible dissected and harvested. In a separate set of experiments, 8-bromo-cGMP (100 gm/kg), a cell-permeable non-hydrolysable analogue of cGMP, was administered daily intravenously (n = 4).

### Histology and immunohistochemistry

The left segment of the mandible was dissected, and the bone cleared of soft tissue before fixing in 4% paraformaldehyde/phosphate buffered saline (PBS), pH 7.4, for 48 h. Tissues were then decalcified in 20% EDTA/PBS, pH 7.4, for 7 days before paraffin processing and embedding. Tissue sections were stained with hematoxylin-eosin for histological evaluation, or used for immunohistochemistry. Antigen retrieval of deparaffinized tissue sections was done by immersing slides in DeCal Retrieval Solution (Biogenex), 20 m, followed by incubation with anti-von Willebrand Factor (1:250), anti-Ki67 (1:100), anti-Runx2 (1:1000), or anti-Osteopontin (1:2500), 16 h at 4°C. Tissue sections were washed and incubated in Biogenex HRP-conjugated universal secondary antibody for 30 m before reacting with diaminobenzidine and counterstaining with hematoxylin.

### Morphometric analysis of regenerating tissue

Slides stained with antibodies to Ki67 and von Willebrand factor were used to assess cell proliferation and vascular indices. Immunostaining in a total of 5 random fields was analyzed, and proliferation index and vascular density was determined by percent cells that stained positive for Ki67 or von Willebrand factor to the total number of hematoxylin-stained nuclei. Immuno-reacted Runx2+ and osteopontin+ cells were similarly measured to evaluate percent osteo-angio-progenitor cell population and percent osteogenic cells. All data were analyzed by Student’st-testto determine p-values.

## Results

### Sildenafil accelerates capillary tube formation *in vitro*

**Figure 1:**
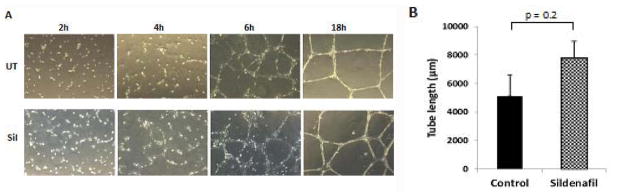
Sildenafil facilitates capillary tube formation *in vitro*. A) HUVEC cells plated on growth factor-reduced matrige l were treated or not with sildenafil (100 nM) and cells were imaged at 2, 4, 6, and 18 h. Quantification of B) total tube length at 4 h. Three random microscopic fields per well were imaged and total tube length quantified by Image J Angiogenesis Analyzer. UT: untreated; Sil: sildenafil-treated.

The effect of sildenafil on capillary tube formation was analyzed after seeding HUVEC on growth factor-reduced matrigel and assessing their organization at regular time intervals. Cells formed branching networks within 6 h, which organized into tube-like structures within 18 h (Figure 1A). The addition of sildenafil hastened the development of cell extensions and the formation of cell-cell connections. At 4 h, the total length of continuous tube connections was greater in sildenafil-treated cells than non-treated counterparts (Figure 1B).

### Sildenafil retards early bone healing

**Figure 2:**
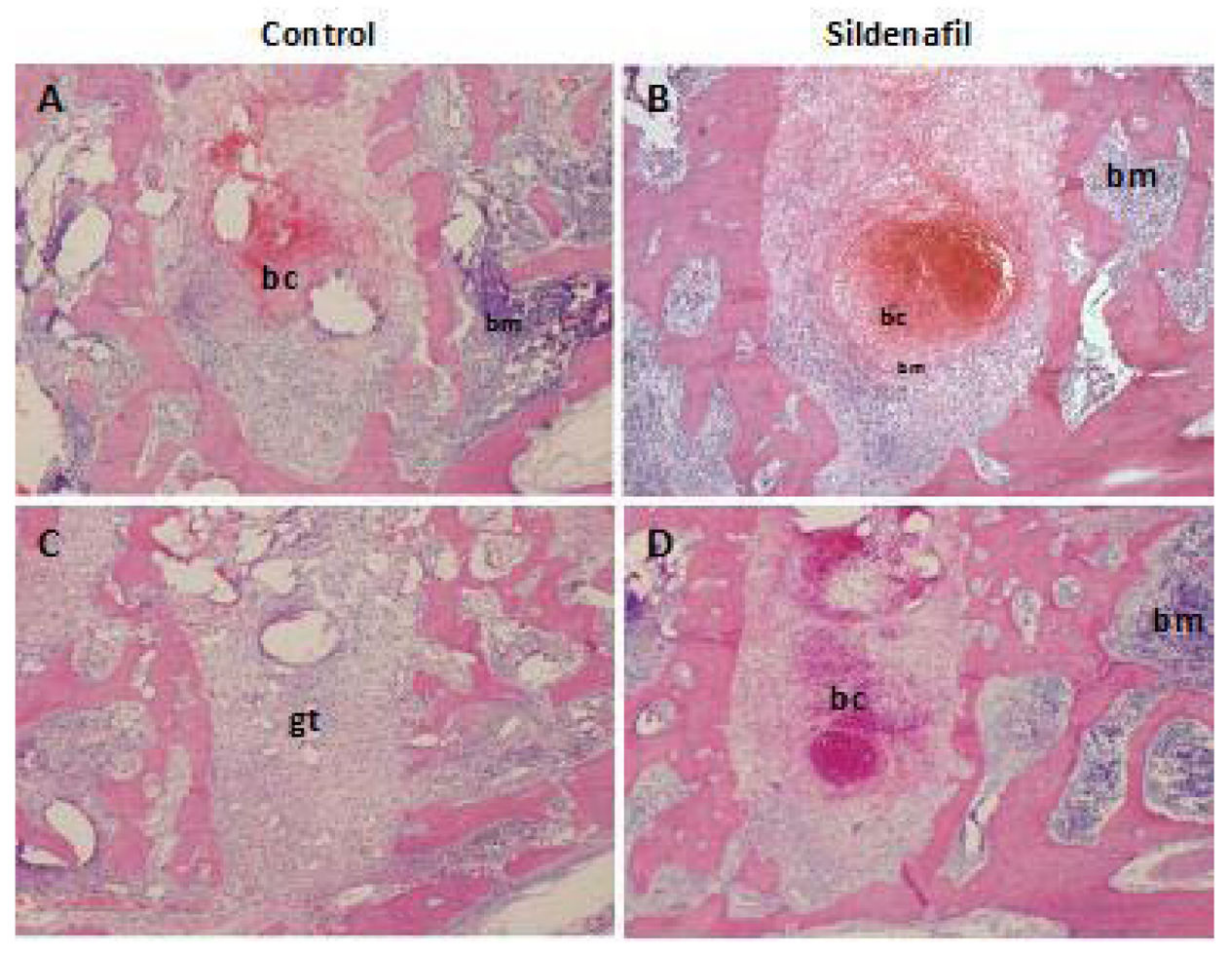
Blood clot resolution is delayed in response to sildenafil. Histological assessment of healing of extraction sockets at A,B) day 2, C,D) day 4, and E,F) day 8 in vehicle treated controls (A,C,E) and sildenafil-treated animals (B,D,F). Images were captured at 40× magnification. bc: blood clot; bm: bone marrow; gt: granulation tissue; ntb: new trabecular bone.

The establishment of blood supply is critical for proper bone healing [5, 21], and sildenafil was shown to accelerate fracture repair in mice [22]. To evaluate whether the drug effect can be recapitulated in alveolar bone healing, we assessed early healing of molar extraction sockets over a period of 8 days. Hematoxylin and eosin stained tissue sections of samples collected on days 2 and 4 following tooth extraction demonstrated a lengthier retention of the sanguine clot in sildenafil-treated compared to vehicle-treated animals (Figure 2A and B). A delay in clot resolution was also observed after administration of non-hydrolysable analog of cGMP, 8-bromo-cGMP (Supplemental Figure 1). A lag in infilling of granulation tissue was a major difference that was observed between groups (Figure 2C and D). However, in contrast to days 2 and 4, healing on day 8 was comparable in drug-treated animals and controls.

### Delay in infiltration of stem/progenitor-rich connective tissue in response to sildenafil

**Figure 3:**
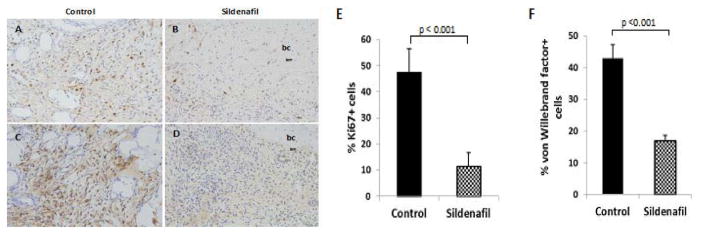
Sildenafil-associated decrease in proliferation and vascular cell densities. Immunohistochemical detection of A,B) Ki-67 and C,D) von Willebrand factor in decalcified, paraffin sections of control and sildenafil-treated animals. E, F) Percent vascular density and proliferating cell population were measured using a ratio of von Willebrand factor+ or Ki-67+ cells to total number of hematoxylin-stained nuclei in 5 random fields each at 200× magnification.

Granulation tissue composed of actively dividing and differentiating stem/progenitor cells is a hallmark of tissue repair and regeneration. Bone tissues harvested at day 4 demonstrated far fewer cells that immuno-reacted with Ki67, a marker of proliferating cells, in response to sildenafil treatment (control: mean±SD, 47.35±9.21; sildenafil: 11.47±5.14; Figure 3A,B and E). Moreover, an assessment of neovasculature by von Willebrand factor immuno-staining demonstrate a decrease in the number of immuno-reactive cells following drug treatment (mean±SD; control: 42.9±4.3; sildenafil: 17.0±1.8; Figure 3C,D and F).

**Figure 4:**
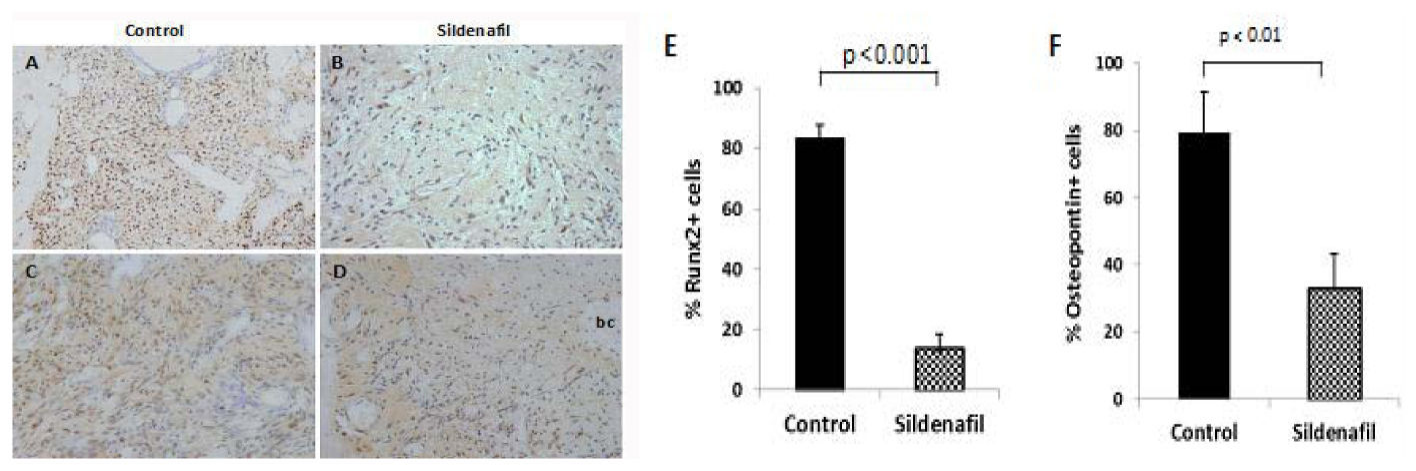
Decreased number of angio- osteo- progenitor cells after sildenafil treatment. Immuno-detection of A,B) Runx2 and C,D) Osteopontin in tissue samples harvested on day 4. Control (A,C) and sildenafil-treated animals (B,D). Percent Runx2+ and Osteopontin+ immuno-stained cells in 5 random fields (200× magnification).

Essential to early osteoblast differentiation is the expression of osteoblast-specific transcription factor, Runt-related Transcription factor 2 (Runx2). Runx2 is highly expressed in immature osteoblasts, but is downregulated during maturation [23]. However, Runx2 is not an exclusive marker of osteogenic cells. It is expressed in endothelial progenitor cells too and has been found to be important for endothelial cell programming [24,25]. To assess the contribution of osteo-angio-progenitor cell population to bone healing, tissue sections were incubated with anti-Runx2. Quantitative morphometric measurement from 5 random fields demonstrated a near 6 fold decrease in cell-staining after drug treatment. The number of Runx2-stained cells in treated and control groups were, control: mean ± SD, 83.18±4.60; sildenafil: mean±SD, 13.77± 4.63 (Figure 4A,B and E).

### Sildenafil retards bone matrix deposition

Runx2 induces the expression of important bone matrix proteins namely, osteopontin, bone sialoprotein, osteocalcin and Col1α1 in immature osteoblasts and mesenchymal progenitors during bone development [23]. To analyze whether the decrease in Runx2-immunoreactive cells translates to a decrease in osteopontin expression after sildenafil treatment, osteopontinimmuno-localization was conducted on tissues collected at day 4. The results demonstrate that, as expected, exposure to the drug results in fewer osteopontin-expressing cells (control: mean±SD, 79.58±11.81; sildenafil: mean±SD, 33.12±10.17; Figure 4C,D and F).

## Discussion

Angiogenesis and the establishment of a functioning vascular bed have been shown to be vital to successful bone repair. Angiogenesis agonists such as vascular endothelial growth factor (VEGF) stimulate bone repair [26], whereas angiogenesis inhibitors suppress bone healing at fracture and implant sites [3–5]. PDE5 is the most active of all cGMP-hydrolyzing PDEs, and it is widely expressed in tissues including bone [27]. In light of previous studies that demonstrated antagonism of PDE5 in promoting neovascularization through augmentation of endothelial cGMP and increase in bone marrow-derived endothelial progenitor cells[27], we anticipated a positive impact of PDE5 inhibitor, sildenafil, on early bone healing. Similar to previous studies, we demonstarte that sildenafil increases HUVEC migration and formation of capillary-like structures on matrigel *in vitro* [13,19]. Its effect on angiogenesis and early bone healing *in vivo*, was assessed in rat tooth extraction sockets. Sequential phases of healing of extraction sockets described before were reproducible in our study[28]. However, the addition of sildenafil retarded blood clot remission and the migration of stem cells into the defect. Since sildenafil principally acts through the cGMP signaling, a delay in resolution of the fibrin clot in cell-permeable 8-bromo-cGMP treated animals implicates cGMP signaling in attenuating clot dissolution. Differentiation monocytes to macrophages or dendritic cells is induced by inflammatory stimuli, and evidence indicates that cyclic nucleotides such as cGMP can impair it [29]. Thus, our results suggest that sildenafil-initiated increase in cGMP has a negative influence on early healing. Alveolar bone formation after tooth extraction occurs by the direct differentiation of mesenchymal stem cells to cells of osteogenic lineage. The important role of Runx2 in osteogenesis has been corroborated by Runx2 knockout mice that have poor skeletal development [30,31]. Runx2 is also expressed in endothelial precursors and it regulates their migration, proliferation, and differentiation [24,32]. Therefore, Runx2 is expressed in cells committed to osteogenic and angiogenic fates, and our finding suggests that the recruitment of stem/progenitor cells is impaired by sildenafil. Earlier studies investigating the effect of sildenafil were focused on its angiogenic effect, but more recently, studies have determined its anti-inflammatory properties [33–36]. Inflammation is a critical initial event in tissue’s response to injury. It signals damage and activates tissue repair mechanisms. The inflammatory response initiated cascade of events plays a significant role bone healing too, and its suppression has been shown to delay healing [37–40]. A recent study examining the effects of sildenafil on long bone fracture repair found that it negatively affects the inflammatory phase of bone healing [41]. Our findings evaluating healing of extraction sockets corroborate the finding that the drug attenuates early events in bone healing. It is suggested that the anti-inflammatory effects of sildenafil suppress blood clot resolution and therefore, the migration of mesenchymal stem cells to the site of injury. Previous studies that analyzed long bone fracture repair at >2 weeks have demonstrated a beneficial effect of sildenafil [22,41], and with no appreciable differences in healing observed at day 8, we suggest that the drug accelerates reparative phase of alveolar bone healing presumably through improved angiogenesis[42,43].

The preservation and maintenance of adequate bone volume after tooth extraction improves the success of prosthetic rehabilitation. Our findings of an early, but transitory, delay in bone healing with sildenafil suggest that a prudent measure would be to avoid the drug in the immediate days following tooth extraction or placement of dental implants.

## Supplementary Material
