# Supplementary material for: Sildenafil Transiently Delays Early Alveolar Healing of Tooth Extraction Sockets
Source: Clin Surg. Author manuscript; Available in PMC 2018 Jun 19. (PMC6006518)
Supplement: Supplementary file 1 [file NIHMS938280-supplement-supplement_1.pdf]

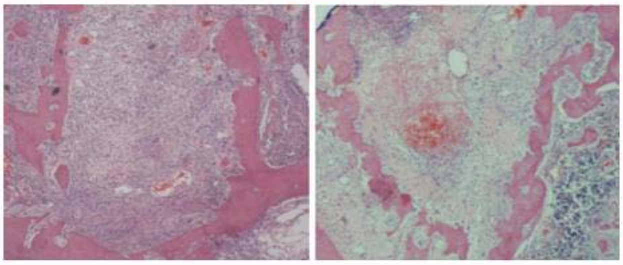

Supplementary Figure 1: Increase in cGMP signaling delays blood clot remission**.** Hematoxylin–eosin staining of bone isolated from A) control and B) 8-bromo-cGMP treated animals 4 days after tooth extraction.

Delay in infiltration of stem/progenitor-rich connective tissue in response to sildenafil

Granulation tissue composed of actively dividing and differentiating stem/progenitor cells is a hallmark of tissue repair and regeneration. Bone tissues harvested at day 4 demonstrated far fewer cells that immuno-reacted with Ki67, a marker of proliferating cells, in response to sildenafil treatment (control: mean±SD, 47.35±9.21; sildenafil: 11.47±5.14; Figure 3A,B and E). Moreover, an assessment of neovasculature by von Willebrand factor immuno-staining demonstrate a decrease in the number of immuno-reactive cells following drug treatment (mean±SD; control: 42.9±4.3; sildenafil: 17.0±1.8; Figure 3C,D and F).

Essential to early osteoblast differentiation is the expression of osteoblast-specific transcription factor, Runt-related Transcription factor 2 (Runx2). Runx2 is highly expressed in immature osteoblasts, but is downregulated during maturation [23]. However, Runx2 is not an exclusive marker of osteogenic cells. It is expressed in endothelial progenitor cells too and has been found to be important for endothelial cell programming [24,25]. To assess the contribution of osteo- angio-progenitor cell population to bone healing, tissue sections were incubated with anti-Runx2. Quantitative morphometric measurement from 5 random fields demonstrated a near 6 fold decrease in cell-staining after drug treatment. The number of Runx2-stained cells in treated and control groups were, control: mean ± SD, 83.18±4.60; sildenafil: mean±SD, 13.77± 4.63 (Figure 4A,B and E).

Sildenafil retards bone matrix deposition

Runx2 induces the expression of important bone matrix proteins namely, osteopontin, bone sialoprotein, osteocalcin and Col1α1 in immature osteoblasts and mesenchymal progenitors during bone development [23]. To analyze whether the decrease in Runx2-immunoreactive cells translates to a decrease in osteopontin expression after sildenafil treatment, osteopontinimmuno-localization was conducted on tissues collected at day 4. The results demonstrate that, as expected, exposure to the drug results in fewer osteopontin-expressing cells (control: mean±SD, 79.58±11.81; sildenafil: mean±SD, 33.12±10.17; Figure 4C,D and F).

Discussion

Angiogenesis and the establishment of a functioning vascular bed have been shown to be vital to successful bone repair. Angiogenesis agonists such as vascular endothelial growth factor (VEGF) stimulate bone repair [26], whereas angiogenesis inhibitors suppress bone healing at fracture and implant sites [3-5]. PDE5 is the most active of all cGMP-hydrolyzing PDEs, and it is widely expressed in tissues including bone [27]. In light of previous studies that demonstrated antagonism of PDE5 in promoting neovascularization through augmentation of endothelial cGMP and increase in bone marrow-derived endothelial progenitor cells[27], we anticipated a positive impact of PDE5 inhibitor, sildenafil, on early bone healing.Similar to previous studies, we demonstarte that sildenafil increases HUVEC migration and formation of capillary-like structures on matrigel*in vitro*[13,19]. Its effect on angiogenesis and early bone healing *in vivo*, was assessed in rat tooth extraction sockets. Sequential phases of healing of extraction sockets described before were reproducible in our study[28]. However, the addition of sildenafil retarded blood clot remission and the migration of stem cells into the defect. Since sildenafil principally acts through the cGMP signaling, a delay in resolution of the fibrin clot in cell-permeable 8-bromo-cGMP treated animals implicates cGMP signaling in attenuating clot dissolution. Differentiation monocytes to macrophages or dendritic cells is induced by inflammatory stimuli, and evidence indicates that cyclic nucleotides such as cGMP can impair it [29].Thus, our results suggest that sildenafil-initiated increase in cGMP has a negative influence on early healing. Alveolar bone formation after tooth extraction occurs by the direct differentiation of mesenchymal stem cells to cells of osteogenic lineage. The important role of Runx2 in osteogenesis has been corroborated by Runx2 knockout mice that have poor skeletal development [30,31]. Runx2 is also expressed in endothelial precursors and it regulates their migration, proliferation, and differentiation [24,32].Therefore, Runx2 is expressed in cells committed to osteogenic and angiogenic fates, and our finding suggests that the recruitment of stem/progenitor cells is impaired by sildenafil. Earlier studies investigating the effect of sildenafil were focused on its angiogenic effect, but more recently, studies have determined its anti-inflammatory properties [33-36]. Inflammation is a critical initial event in tissue's response to injury. It signals damage and activates tissue repair mechanisms. The inflammatory response initiated cascade of events plays a significant role bone healing too, and its suppression has been shown to delay healing [37-40]. A recent study examining the effects of sildenafil on long bone fracture repair found that it negatively affects the inflammatory phase of bone healing [41].Our findings evaluating healing of extraction sockets corroborate the finding that the drug attenuates early events in bone healing. It is suggested that the anti-inflammatory effects of sildenafil suppress blood clot resolution and therefore, the migration of mesenchymal stem cells to the site of injury. Previous studies that analyzed long bone fracture repair at >2 weeks have demonstrated a beneficial effect of sildenafil [22,41], and with no appreciable differences in healing observed at day 8, we suggest that the drug accelerates reparative phase of alveolar bone healing presumably through improved angiogenesis[42,43].

The preservation and maintenance of adequate bone volume after tooth extraction improves the success of prosthetic rehabilitation. Our findings of an early, but transitory, delay in bone healing with sildenafil suggest that a prudent measure would be to avoid the drug in the immediate days following tooth extraction or placement of dental implants.

Acknowledgement

The authors thank SenthilnathanPalaniyandi for his contribution during the early stages of the capillary tube formation study. The research was supported by funds from the American Cancer Society, National Institutes of Health: NIH/NCI R21CA173162, and the FeistWeiller Cancer Center.
